# Different Neurogenic Potential in the Subnuclei of the Postnatal Rat Cochlear Nucleus

**DOI:** 10.1155/2021/8871308

**Published:** 2021-04-05

**Authors:** Johannes Voelker, Jonas Engert, Christine Voelker, Linda Bieniussa, Philipp Schendzielorz, Rudolf Hagen, Kristen Rak

**Affiliations:** Department of Oto-Rhino-Laryngology, Plastic, Aesthetic and Reconstructive Head and Neck Surgery and the Comprehensive Hearing Center, University of Wuerzburg, Germany

## Abstract

In patients suffering from hearing loss, the reduced or absent neural input induces morphological changes in the cochlear nucleus (CN). Neural stem cells have recently been identified in this first auditory relay. Afferent nerve signals and their impact on the immanent neural stem and progenitor cells already impinge upon the survival of early postnatal cells within the CN. This auditory brainstem nucleus consists of three different subnuclei: the anteroventral cochlear nucleus (AVCN), the posteroventral cochlear nucleus (PVCN), and the dorsal cochlear nucleus (DCN). Since these subdivisions differ ontogenetically and physiologically, the question arose whether regional differences exist in the neurogenic niche. CN from postnatal day nine Sprague-Dawley rats were microscopically dissected into their subnuclei and cultivated in vitro as free-floating cell cultures and as whole-mount organ cultures. In addition to cell quantifications, immunocytological and immunohistological studies of the propagated cells and organ preparations were performed. The PVCN part showed the highest mitotic potential, while the AVCN and DCN had comparable activity. Specific stem cell markers and the ability to differentiate into cells of the neural lineage were detected in all three compartments. The present study shows that in all subnuclei of rat CN, there is a postnatal neural stem cell niche, which, however, differs significantly in its potential. The results can be explained by the origin from different regions in the rhombic lip, the species, and the various analysis techniques applied. In conclusion, the presented results provide further insight into the neurogenic potential of the CN, which may prove beneficial for the development of new regenerative strategies for hearing loss.

## 1. Introduction

**Figure 1:**
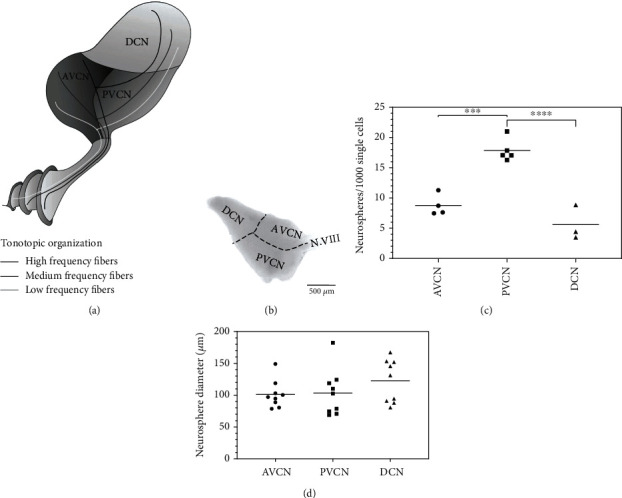
The dissected CN subnuclei form neurospheres in free-floating cell cultures in NSC medium. (a) Schematic overview of the topographical arrangement of the CN subnuclei, the auditory nerve, and the cochlea (AVCN = anteroventral cochlear nucleus; PVCN = posteroventral cochlear nucleus; DCN = dorsal cochlear nucleus). The tonotopic organization of the N.VIII fibers is maintained in all subnuclei. (b) Microscopic reflected light image of a PND 9 CN preparation and labeling of the subnuclei and the cochlear nerve (N.VIII) before dissection. (c) Dissociated cells of the CN subnuclei form neurospheres in free-floating cell cultures under the influence of EGF and bFGF. The quantitative evaluation after 30 days showed that the PVCN has the significantly greatest potential for the formation of neurospheres with 17.9 ± 1.9 (*n* = 5; mean ± SD) spheres per 1000 initially cultured single cells. (d) Evaluation of the neurosphere diameters after 4 weeks in free-floating cultures. The diameters averaged 101.2-122.6 *μ*m. No significant differences were found between the subnuclei. Box plots show the median with the upper and lower quartiles, and whiskers mark the upper and lower maximum values; asterisks indicate the significance level: ^∗^*p* < 0.05, ^∗∗^*p* < 0.005, ^∗∗∗^*p* < 0.001, and ^∗∗∗∗^*p* < 0.0001.

In patients suffering from hearing loss, cochlear hair cells and consecutively the spiral ganglion neurons are subject to degeneration. This pathomechanism affects the transduction process in the inner ear and the ascending central auditory pathway in transmission to the auditory cortex. The lack of neural input results in a morphological change in the auditory brainstem nuclei, particularly in the cochlear nucleus (CN) [1, 2]. The CN is located on the lateral side of the mammalian brainstem and is the first relay station for acoustically derived neural input from the inner ear. The cochlear nerve accesses the CN between its antero- and posteroventral part (Figures 1(a) and 1(b)). Nerve fibers are in contact with all three segments of the CN [3]. Connections from the primary auditory neurons, the spiral ganglion cells, are tonotopically organized. In the CN, the secondary auditory neurons arise, which transmit the acoustic information to higher levels in the brainstem, the olivary complex, the nucleus of the lateral lemniscus, and the inferior colliculus. The CN consists of different subnuclei: the ventral cochlear nucleus (VCN) and the dorsal cochlear nucleus (DCN). The VCN can then be further divided into the posteroventral cochlear nucleus (PVCN) and the anteroventral cochlear nucleus (AVCN). Different tonotopically organized fibers reach the CN from the similarly organized spiral ganglion cells. From the subnuclei, various projections lead to further central auditory nuclei, which are involved in aspects of signal processing [4]. The subnuclei each have specific electrophysiological functions. Certain areas of the CN perform the frequency coding of the auditory input, time measurements of the signal, and the sound localization [5, 6].

The functional aspects of adult neurogenesis have been discussed since their first description [7]. They could potentially promote endogenous remodeling processes in the context of neuroplasticity or be explicitly used for regenerative therapies for the spiral ganglion cells of the cochlea and the auditory pathway [8–10]. *In vitro* analyses have shown that stem cell-derived neurons can regenerate lost synaptic connections from spiral ganglion and CN neurons [11]. Following differentiation, induced pluripotent stem cells were able to form synapses with the denervated sensory epithelium of the cochlea and regenerate the lost axonal connections *in vitro* [12]. It is also described that, in addition to the reduced or missing neural input in sensorineural hearing loss, neural progenitor cells (NPC) play a role in the CN to degenerate under signal deprivation [13]. Neural stem cells (NSCs) have recently been identified in the postnatal CN. The cells have all basic features of neural stem cells, particularly the capacity of mitotic self-renewal as well as the differentiation into neural progenitor cells and all cells of the neural lineage [14]. In addition to the early postnatal period in which neurogenesis was described within the CN [15], previous studies demonstrated a low neurogenic potential up to postnatal day (PND) 40. It persists until adulthood, which was shown by an ongoing capacity of neurosphere formation, BrdU incorporation, and detection of NSCs by specific markers [16]. The NSCs of the CN were able to differentiate after transplantation into neural tissue in all cells of the neural lineage [17].

While it is already known that the embryonic development of the CN subnuclei occurs in tonotopic specificity [18] and that region-specific pathologies exist in the adult organism [19–24], it has not yet been clarified whether there is a postnatal neurogenic potential in all compartments. The question arose whether regional differences exist, which could play a role in regeneration after damage to the auditory pathway. To investigate this question, the subnuclei of the CN were dissected and analyzed for their specific neurogenic potential in PND 9 Sprague-Dawley rats. The compartments were examined separately immunohistologically. Whole-mount cultures and free-floating cell cultures were generated from dissociated preparations. In addition to immunocytological analyses and quantitative evaluations, cell cycle analyses were carried out using bromodeoxyuridine (BrdU).

## 2. Material and Methods

### 2.1. Animals and Tissue Preparations

All procedures were carried out following the guidelines established for experimental methods by German law (§8, German Animal Protection Act). Postnatal day 9 Sprague-Dawley rats (Charles River®) were euthanized by cervical dislocation and decapitation. The skull was opened midsagittally, and the bone removed. After careful dissection of the cranial nerves, the brain was lifted out and transferred into 35 mm Petri dishes in a 5°C cold phosphate-buffered saline solution (DPBS, 0.05 M, PAA Laboratories®). Using a stereomicroscope (ZEISS®, Stemi 508), the cerebrum and cerebellum were separated at 5x magnification, and the brainstem was freed from meningeal tissue and blood vessels. After identifying the CN, it was bluntly dissected with #5/45 preparation forceps (Dumont®). After dissection of the CN, the stereomicroscope was switched from reflected light mode to transmitted light high-contrast mode, so that the fine structural boundaries of the subnuclei became faintly visible. The separation of the compartments was then carried out with a scalpel no. 13 at 25x magnification. Primarily, a cut was made in the middle, orthogonal to the longitudinal axis, to separate the ventral and dorsal parts. Another cut followed this through the ventral part along the longitudinal axis in an extension of the inserting N.VIII (Figure 1(b)). Subsequently, the compartments were processed individually.

### 2.2. Cell Culture and Neurosphere Assay

The dissociation of neural tissue was performed enzymatically in Accutase (Gibco®, Thermo Fisher Scientific®) for 30 min at 37°C in a ThermoMixer® (Eppendorf®). The suspension was triturated every 10 min with a 500 *μ*l pipette. Then, it was centrifuged (1000 rpm, 5 min), and the pellet suspended in neural stem cell medium (NSC medium), containing serum free Neurobasal® (Thermo Fisher Scientific®), 1% GlutaMAX supplement (Invitrogen®), B27 supplement without retinoic acid (Invitrogen®), and 1% penicillin/streptomycin (Invitrogen®). The recombinant murine growth factors EGF (10 mg/ml) (PeproTech®) and bFGF/FGF-2 (10 ng/ml) (PeproTech®) were added. The number of cells in the individual samples was determined using the Neubauer hemocytometer (ZK06, Hartenstein®). Vital cells were determined by staining with trypan blue (0.4%, #93595, Sigma-Aldrich®). Free-floating cell cultures were generated in hydrophobic cell culture flasks (T25, CELLSTAR®, filter top, 25 cm^2^, Greiner Bio-One®) at 37°C and 5% CO_2_. The number of primary spheres was determined after 4 weeks in culture and recalculated for about 1000 cultured cells.

For analyses, neurospheres were carefully aspirated from the free-floating cell cultures with 5 ml autopipettes (accu-jet pro, Brand®) and plated onto glass coverslips (78.5 mm^2^, Hartenstein, precoated with poly-D-lysine (100 *μ*g/ml, SERVA Electrophoresis®) and laminin-1 (10 *μ*g/ml, BD Biosciences®)). The spheres were cultivated in 4-well dishes (Greiner Bio-One®), each with 100 *μ*l of NSC medium per well. The integrity of the plated spheres was checked with an inverted transmitted light microscope (Leica® DMI-8). Cultures were then incubated at 37°C/5% CO_2_ for the intended period. The medium was changed every two days to fresh NSC medium after careful aspiration of the used medium with Pasteur pipettes.

### 2.3. Plating of Single Cells and Induction of Cell Differentiation

Cell culture suspension was removed with a 5 ml accu-jet pipette (Brand®) from the free-floating neurosphere cultures and then centrifuged at 1000 rpm for 2 min. The medium supernatant was then aspirated from the pellet, and 100 *μ*l of Accutase (Gibco®, Thermo Fisher Scientific®) was added. The suspension was incubated for 15 min at 37°C and 500 rpm in a ThermoMixer® (Eppendorf®); the suspension was triturated every 5 min with the 200 *μ*l pipette. The samples were then centrifuged at 1000 rpm for 2 min, the Accutase was suctioned off, and the cell pellet was resuspended with cell medium. The single cells were either transferred as new passages into free-floating cultures or plated.

For cell differentiation, the individual cells were plated in differentiation (DIF) medium consisting of Neurobasal (Thermo Fisher Scientific®), GlutaMAX (Invitrogen®), and B27 with retinoic acid (Invitrogen®). The 2-D culture was carried out on glass coverslips coated with laminin-1 (1 : 100 in 0.05 M D-PBS) and poly-D-lysine (PDL; 1 : 100 in 0.05 M D-PBS) with a density of 100 cells/mm^2^. The culture was carried out in 4 wells for a total of 8 days at 37°C/5% CO_2_. A medium change was carried out every two days.

### 2.4. Fixation and Immunocytochemistry

After the experiments were completed, single cells and spheres were fixed with a 4% paraformaldehyde solution (PFA in 0.1 M PBS) for 30 min and finally for 5 min in acetone. The nonspecific binding sites were blocked with a 10% bovine serum albumin solution (BSA, A9418, Sigma-Aldrich®) in 0.1 M PBS. For immunocytochemistry, the preparations were incubated with the following primary antibodies at 5°C for 12 h in 1% BSA solution and 0.1 M PBS: mouse monoclonal against Atoh1 (1 : 1000; Ab27667, Abcam), mouse monoclonal against BrdU (5-bromo-2′-deoxyuridine) (1 : 600; #05-633, Millipore®), mouse monoclonal against *β*-tubulin (1 : 1000; #TS293, Sigma-Aldrich), mouse monoclonal against *β*-III-tubulin (1 : 1000; #Ab7751, Abcam®), rabbit polyclonal against *β*-III-tubulin (1 : 2000; #Ab18207, Abcam®), rabbit polyclonal against doublecortin (DCX) (1 : 1000; #Ab18723, Abcam®), mouse monoclonal against glial fibrillary acidic protein (GFAP) (1 : 1000; #MAB360, Millipore®), rabbit polyclonal against myelin basic protein (MBP) (1 : 800; #M3821, Sigma-Aldrich®), mouse monoclonal against nestin (1 : 800; #MAB353, Millipore®), and rabbit polyclonal against Sox-2 (1 : 2000; #Ab97959, Abcam®). After rinsing three times with 0.1 M PBS solution, the secondary antibodies were incubated, coupled to Alexa Fluor A488 and A555 (1 : 1000, #A11001, #A11008, Thermo Fisher) with 5 *μ*g/ml DAPI (1 : 5000, D9542, Sigma-Aldrich®) for 1 h in a 1% BSA solution in 0.1 M PBS. Finally, three rinsing steps were carried out again in 0.1 M PBS, and the glass coverslips were embedded on glass slides with Mowiol (#4-88, Sigma-Aldrich®). The storage took place at 5°C in light-protected folders.

### 2.5. Whole-Mount Preparation and BrdU Assay

After preparation and sectioning of the CN subnuclei, these were carefully picked up with spring steel tweezers (100/8 mm, Ehlert & Partner®) and placed on semiporous (0.4 *μ*m) membranes (Greiner Bio-One®) in 12-well dishes (Greiner Bio-One®). One drop of cell medium was added on top. NSC medium containing 5-bromo-2′-deoxyuridine (BrdU) (10 *μ*M) was added until the level reached the membrane. The incubation was carried out at 37°C/5% CO_2_. For tissue fixation, a 4% PFA solution was first added to the cell culture medium in a 1 : 1*w* ratio after 48 h and then removed. The further fixation was carried out for 2 h in a 4% PFA solution in 0.1 M PBS. After fixation, the tissue was transferred to 0.1 M PBS solution and stored at 5°C or immediately processed for immunohistochemistry.

### 2.6. Histological Sectioning and Immunohistochemistry

After fixation, the tissue was incubated in 30% saccharose overnight, cryoprotected in Tissue-Tek O.C.T. (Sakura®), and frozen in liquid nitrogen. Sections of 20 *μ*m were cut by using a cryostat (Leica®, CM3050 S), mounted on superfrost slides (Hartenstein®), and stored (-20°C) until further analysis. For immunofluorescence staining, sections were postfixed in 4% PFA for 5 min, rinsed in TBS-T three times, and blocked with 10% BSA in 0.3% Triton X-100 (Sigma-Aldrich®) for 1 h. Primary antibodies were incubated in 1% BSA in 0.3% Triton X-100 for 48 h at the following concentrations: mouse monoclonal against BrdU (1 : 25) (Millipore®), mouse monoclonal against nestin (1 : 500, Millipore®), rabbit polyclonal against Atoh1 (1 : 100, Santa Cruz®), rabbit polyclonal against Sox-2 (1 : 100, Abcam®), and rabbit polyclonal against doublecortin (DCX) (1 : 1000; #Ab18723, Abcam®). The sections were then rinsed three times with TBS-T and incubated for 1 h in 1% BSA in TBS-T with goat anti-rabbit or goat anti-mouse secondary antibody coupled to Alexa Fluor A488 and A555 (1 : 1000, #A11001, #A11008, Thermo Fisher) and 5 *μ*g/ml DAPI (1 : 5000, D9542, Sigma-Aldrich®). After washing three times with TBS-T, the sections were embedded on glass slides in Mowiol (#4-88, Sigma-Aldrich®).

### 2.7. Flow Cytometry and Cell Cycle Analysis

For cell cycle analysis, 1 ml of cell suspension was taken per test and centrifuged at 1000 rpm for 5 min. After the supernatant had been removed by suction, the cell suspension was dissociated by using Accutase® for 15 min at 37°C. After centrifugation, the pellet was resuspended in 500 *μ*l PBS 0.1 M and 1000 *μ*l 70% ethanol was added for permeabilization for 2 hours at 4°C (Fisher Bioreagents®, #BP8201-1). After centrifugation at 1000 rpm for 5 min, the supernatant was sucked off, and the pellet was resuspended in 500 *μ*l PBS (0.1 M) in 0.25% Triton X-100 for 15 min at room temperature. After centrifuging and washing in 500 *μ*l PBS, 100 *μ*l anti-phopho-histone-H3 (pHH3) antibody solution was added (1 : 1000 in 1x PBS+1% BSA; rabbit mAb #3377, Cell Signaling Technologies®). Incubation took place overnight at 4°C. On the following day, the first antibody solution was centrifuged off at 1000 rpm, and the pellet was resuspended in 100 *μ*l of the second antibody solution: anti-rabbit Alexa 635 (goat anti-rabbit IgG secondary antibody-Alexa Fluor 635 (Thermo Fisher Scientific®, #A-31577)). After 30 min incubation at room temperature in the dark box, the pellet was diluted with 1000 *μ*l PBS and the supernatant was centrifuged off afterward. 1000 *μ*l of PI/RNase Staining Solution (BD Biosciences®, #550825) was then added, and the pellet was resuspended therein and transferred into FACS-suitable round-bottom tubes (Falcon™, Thermo Fisher Scientific®). After an incubation time of 15 min in the dark, the analysis was carried out with a BD FACSCanto® Flow Cytometer. The measurements and the data evaluation were carried out with the BD FACSDiva software V 5.0.3 (BD Biosciences®).

### 2.8. Digital Images and Picture Analysis

Digital images of the cell cultures and preparations were taken with a Leica® DMI8 fluorescence microscope and Leica Application Suite X software v3.0.1 (Leica®). To quantify the number of neurospheres, all culture flasks were scanned using the transmitted light technique in tile scan mode. The digital images of microscopic images were exported directly from the Leica Application Suite software in the uncompressed TIFF format. The final image composition was done with Adobe® InDesign CC 2020 v15.0.2 software. The tissue sections were analyzed with an Olympus® Fluoview FV3000 confocal laser scanning microscope and exported with Fiji/ImageJ V2.0.0 software [25].

### 2.9. Statistical Analysis

All data were compiled using Microsoft Excel 2019 V16.34 spreadsheets and statistically analyzed with GraphPad® Prism 8.2.0 software. First, a column analysis (D'Agostino-Pearson omnibus normality test) was performed to determine whether a Gaussian normal distribution of the data was present. Subsequently, data were analyzed using the ordinary one-way ANOVA test followed by the Tukey multiple comparison test. A *p* value < 0.05 was considered to be statistically significant. Reproducible results were obtained from three or more samples. If the data followed a Gaussian normal distribution, mean and standard error of the mean (SEM) are displayed, whereas without Gaussian normal distribution, mean and standard deviation (SD) are displayed.

## 3. Results

To investigate the neurogenic potential in the CN subnuclei and whether it is similar or significantly different in all regions, a systematic in vitro analysis was done. After the subnuclei had been dissected, a neurosphere assay was carried out. The cells propagated here were examined immunocytochemically. The essential criteria for neural stem cells were characterized. To compare the results from the neurosphere assay with the neurogenic potential in the tissue of subnuclei, whole-mount organ cultures were carried out and examined immunohistochemically.

### 3.1. The CN Subnuclei Show an Individually Different Capacity for Neurosphere Formation

The dissociated cells of the anteroventral portion of the CN (AVCN) formed 8.77 ± 1.76 neurospheres/1000 viable cells (*n* = 5) within 30 days. The posteroventral portion (PVCN) formed 17.85 ± 1.85 neurospheres/1000 viable cells (*n* = 5), and the dorsal portion (DCN) yielded 5.58 ± 2.87 neurospheres/1000 viable cells (*n* = 5). The peak potential for the formation of neurospheres was found in the PVCN (AVCN vs. PVCN: *p* < 0.001, PVCN vs. DCN: *p* < 0.0001). There was no significant difference between cells of the AVCN and DCN (Figure 1(c)).

In addition to the rate of neurospheres formed, their diameter was also determined by light microscopy after the expansion phase. AVCN cells formed neurospheres with diameters of 101.2 ± 21.68 *μ*m on average, over 30 days. The PVCN formed spheres measuring 103.5 ± 36.47 *μ*m, and the DCN formed spheres with average diameters of 122.6 ± 33.8 *μ*m (*n* = 5). No significant differences in the individual subnuclei were determined here (Figure 1(d)).

### 3.2. Neurospheres of All CN Subnuclei Show Neural Progenitor Cell Markers

**Figure 2:**
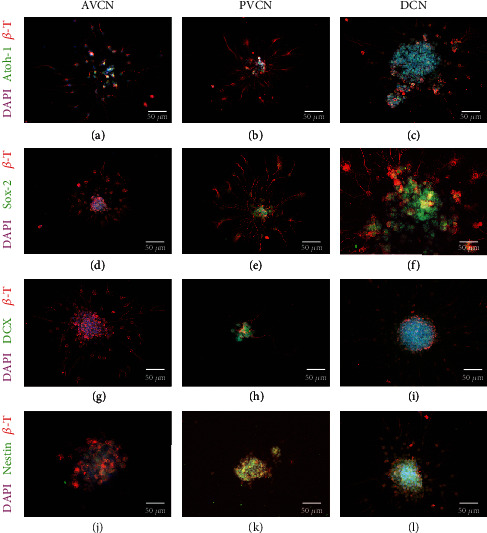
Neurospheres from all CN subnuclei contain neural progenitor cells. These spheres adhered to glass coverslips, coated with poly-d-lysine and laminin-1. After 48 h in NSC medium, these showed centrifugally outgrowing branches and emigrating cells, stained for *β*-tubulin. Cells in the neurospheres of all subnuclei showed a positive staining for the transcription factors Atoh-1 (a–c) and Sox-2 (d–f). (g–i) Neural stem cells were identified by the markers DCX (doublecortin) and nestin (j–l). Cell nuclei were stained with DAPI.

After 48 hours, the spheres formed extensions on the surfaces in 2-D cultures, and individual cells emigrated centrifugally. These structures were visualized with *β*-tubulin staining (Figures 2(a)–2(l)). The transcription factor Atoh-1, a derivation marker of neural stem cells of the auditory pathway, was detected in the immunocytochemical staining (Figures 2(a)–2(c)). Neurospheres from all CN subnuclei expressed this marker as well as the progenitor cell marker Sox-2 (Figures 2(d)–2(f)). Furthermore, the neuronal migration protein doublecortin (DCX) (Figures 2(g)–2(i)) and the progenitor cell marker nestin were detected in cells of the neurospheres of all CN subdivisions (Figures 2(j)–2(l)).

To further quantify the ability to form progenitor cells, neurospheres of the individual subdivisions were dissociated into single cells and plated. After 24 hours in NSC medium, the progenitor cell markers already detected in neurospheres were quantified in single cells and compared to the absolute cell numbers of viable cells, stained with *β*-tubulin. Atoh-1 was stained positively in AVCN cells in 58.28 ± 22.98%, in PVCN cells in 49.92 ± 18.4%, and DCN cells in 81.59 ± 15.09% (*n* = 3; mean ± SD). There were no significant differences between the CN subnuclei. Sox-2 was stained positively in AVCN cells in 59.55 ± 12.78%, in PVCN cells in 28.2 ± 20.52%, and DCN cells in 74.22 ± 7.13% (*n* = 3; mean ± SD). There were significant differences concerning DCN vs. PVCN (*p* < 0.05), and the lowest proportion of cells in the PVCN is Sox-2-positive. DCX was stained positive in AVCN cells in 54 ± 8.47%, in PVCN cells in 54.82 ± 6.02%, and in DCN cells in 57.88 ± 3.98% (*n* = 3; mean ± SD). There were no significant differences between the CN subnuclei. Nestin was positively stained in AVCN cells in 57.9 ± 13.14%, in PVCN cells in 84.42 ± 2.12%, and DCN cells in 37.11 ± 14.85% (*n* = 3; mean ± SD). There were significant differences in PVCN vs. DCN (*p* < 0.005). The largest proportion of nestin-positive cells is detectable in the PVCN.

### 3.3. CN Progenitor Cells of All Subnuclei Can Differentiate

**Figure 3:**
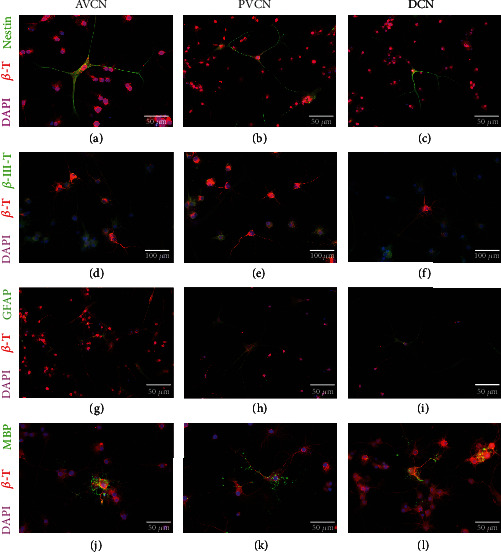
Neural stem cells of all CN subnuclei can differentiate into the cell forms of the neuroectodermal line. (a–c) Undifferentiated cells showed a positive staining for the progenitor cell marker nestin. (d–f) Neuronally differentiated cells were identified by *β*-III-tubulin staining (red). These cells had slim, spindle-shaped, neuron-typical somata with bi- and multipolar formations. (g–i) Astrocytes were identified by GFAP staining (glial fibrillary acidic protein). These cells showed an astrocyte-typical stellate-shaped formation with multipolar branches. (j–l) Oligodendrocytes were stained with MBP (myelin basic protein). These showed a beginning myelinization of the peripheral processes, while the still unmyelinated cell bodies were stained *β*-tubulin (*β*-T) positive. Cell nuclei were stained with DAPI.

Cultures of all three CN subnuclei contained single cells in the immunocytochemical analysis which were positive for the progenitor cell marker nestin (Figures 3(a)–3(c)). Although these multipolar cells showed morphological signs of differentiation, they differed significantly from other *β*-tubulin-labeled cells, which formed widely branched networks.

Differentiated morphologically diverse cell forms were found in the cultures of all three CN subnuclei. Neuronally differentiated cells with spindle-shaped somata and bipolar or multipolar processes were identified by *β*-III-tubulin (Figures 3(d)–3(f)). Star-shaped branched cells, which stand out morphologically clearly from the surrounding bipolar neuronally differentiated cells, expressed GFAP (glial fibrillary acidic protein). These were identified as astrocytes in cultures of all three subnuclei (Figures 3(g)–3(i)). Furthermore, tree-branched cells were found in the DIF cultures of all three subnuclei, making peripheral contact with the neuron-differentiated cells with their branches. The onset of myelination of their cell processes was shown by the oligodendrocyte-specific marker MBP (myelin basic protein) (Figures 3(j)–3(l)).

**Figure 4:**
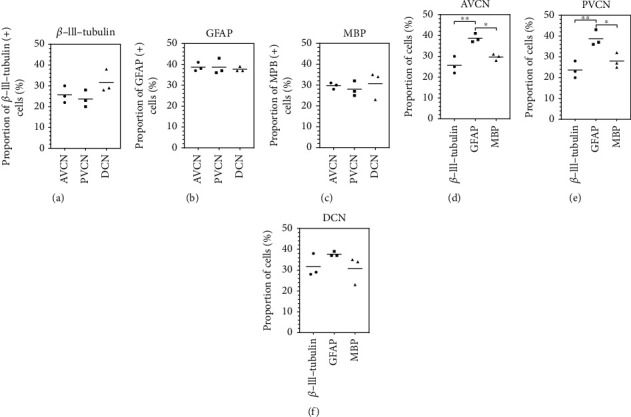
Propagated stem cells of all subnuclei express markers of the neuroectodermal lines after an eight-day differentiation phase. It was examined whether there are relevant differences in differentiation in cultures of the different regions. The marker *β*-III-tubulin identifies neuronally differentiated cells. Astrocytes show expression of GFAP, and oligodendrocytes can be identified immunohistochemically by MBP. (a) AVCN, PVCN, and DCN showed, on average, 26-32% *β*-III-tubulin-positive neurons (mean; *n* = 3). (b) The majority of the cells were glial differentiated and GFAP positively stained (38-39%) (mean; *n* = 3). (c) MBP-positive labeled oligodendrocytes were present in 28-31% of the cells of the subnuclei (mean; *n* = 3). There were no significant differences in the rates of neural stem cell differentiation between the CN subnuclei. (d–f) Intraindividual analysis of the relations of differentiation markers. Significant variances in differentiation were found in progenitor cells from AVCN and PVCN. Most of the cells expressed the glial cell marker GFAP, followed by oligodendrocytes (MBP). The smallest part of the cells showed neuronal differentiation with *β*-III-tubulin expression. The same tendency emerged in the area of the DCN. Box plots show the median with the upper and lower quartiles, and whiskers mark the upper and lower maximum values; asterisks indicate the significance level: ^∗^*p* < 0.05, ^∗∗^*p* < 0.005, ^∗∗∗^*p* < 0.001, and ^∗∗∗∗^*p* < 0.0001.

Furthermore, it was examined whether the subnuclei show relevant differences in the relative stem cell fates. Therefore, the single cells were quantified by the differentiation markers to the absolute number of cells (*β*-tubulin-positive cells). Progenitor cells of the AVCN developed 25.67 ± 4.04%*β*-III-tubulin (+) neuronally differentiated cells after an eight-day differentiation phase. The PVCN showed 23.67 ± 4.0%*β*-III-tubulin (+) cells, and the DCN showed 31.67 ± 5.5% (*n* = 3; mean ± SD). GFAP-positive glial cells were detected in AVCN in 38.67 ± 2.08%, in PVCN in 39 ± 3.8%, and in DCN in 37.7 ± 1.2% (*n* = 3; mean ± SD). MBP-positive glial cells with an oligodendrocyte morphology were detected in AVCN in 29.67 ± 1.53%, in PVCN in 28 ± 3.6%, and in DCN in 30.7 ± 6.7% (*n* = 3; mean ± SD) (Figures 4(a)–4(c)).

To investigate whether the differentiation of progenitor cells of the CN subnuclei showed intraindividual or interindividual differences, the statistical analysis of these two modalities was juxtaposed (Figures 4(a)–4(c) vs. Figures 4(d)–4(f)). The ability to differentiate neural progenitor cells of all CN subnuclei was demonstrated without showing any significant interindividual differences in potential (Figures 4(a)–4(c)). In contrast, the intraindividual analyses showed that in the AVCN and PVCN, the significant majority of the cells were differentiated astrocytically, followed by neural cells and followed by oligodendrocytes. This tendency was also evident in the DCN (Figures 4(d) and 4(e)).

### 3.4. Neural Stem Cell Markers Are Expressed in Tissue Sections of the CN Subnuclei

Since cardinal stem cell criteria were shown in all CN subdivisions by the cell culture assay, the question arose whether they can also be detected in the CN tissue itself. In addition, mitotic activity should be examined by BrdU incorporation. Therefore, the dissected subnuclei were incubated as whole-mount organ cultures on Transwell® membranes for 48 hours.

**Figure 5:**
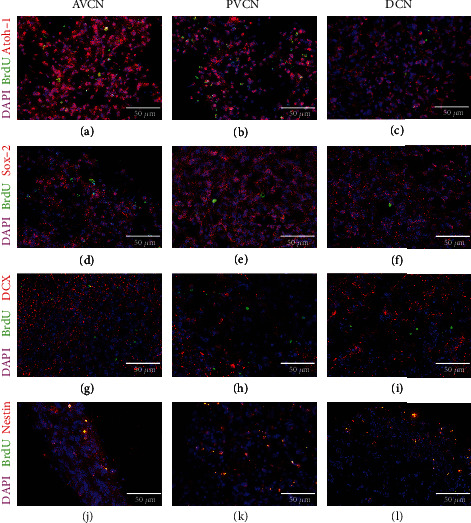
Neurogenesis in the CN subnuclei. Preparations from PND 9 animals were incubated as whole-mount organ cultures for 48 h in stem cell medium and the S phase marker BrdU. The immunohistological analysis showed the expression of neural stem cell markers and mitotic activity of individual cells. The transcription factors Atoh-1 and Sox-2 as well as the neural stem cell markers DCX and nestin were detected in preparations from all subnuclei. All whole-mount organ cultures showed a BrdU uptake of individual cells after an incubation period of 48 h. Cell nuclei were stained with DAPI.

The neurogenic transcription factors Atoh-1 and Sox-2 were detectable in sections of the AVCN, PVCN, and DCN (Figures 5(a)–5(f)). The neuronal migration protein, doublecortin (DCX), was also shown in all subdivisions (Figures 5(g)–5(i)). Nestin also was stained immunohistochemically in all three CN subnuclei (Figures 5(j)–5(l)). The neurogenic activity was visualized in all subnuclei by the S phase marker bromodeoxyuridine (BrdU) (Figures 5(a)–5(l)).

### 3.5. Progenitor Cells of CN Subnuclei Show Different Mitotic Activities in the BrdU Assay

**Figure 6:**
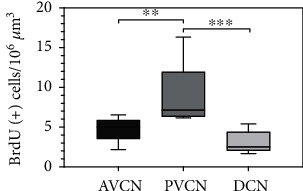
The whole-mount organ cultures of CN subnuclei showed a different mitotic capacity in the BrdU assay. A positive BrdU staining of individual cells was demonstrated in all examined preparations. The AVCN had an average of 4.7 ± 1.5 BrdU (+) cells/10^6^ *μ*m^3^. The PVCN had with 9 ± 3.9 BrdU (+) cells significantly the highest mitotic activity (mean ± SD); the DCN contained 3.1 ± 1.4 BrdU (+) cells/10^6^ *μ*m^3^ (*n* = 9). Box plots demonstrate the median with the upper and lower quartiles, and whiskers mark the upper and lower maximum values; asterisks indicate the significance level: ^∗^*p* < 0.05, ^∗∗^*p* < 0.005, ^∗∗∗^*p* < 0.001, and ^∗∗∗∗^*p* < 0.0001.

To investigate the potential of cell division as a stem cell characteristic, the different parts of the CN were cultivated as whole-mount organ cultures with BrdU for 48 h. Subsequently, the quantification of BrdU (+) cells in the various sections was performed. After incubation for 48 h, 4.64 ± 1.47 BrdU (+) cells/10^6^*μ*m^3^ (*n* = 9; mean ± SD) were found in the whole-mount preparations of the AVCN, 8.22 ± 3.4 BrdU (+) cells/10^6^ *μ*m^3^ were found in the PVCN (*n* = 9; mean ± SD), and 3.1 BrdU(+) cells/10^6^ *μ*m^3^ (*n* = 9; mean ± SD) were found in the DCN. The PVCN showed a significant higher potential for proliferation compared to the AVCN and DCN. No significant difference was measurable between AVCN and DCN (AVCN vs. PVCN: *p* < 0.01, PVCN vs. DCN: *p* < 0.001) (Figure 6).

### 3.6. Flow Cytometric Cell Cycle Analyses Show Different Proportions of Progenitor Cell Division

**Figure 7:**
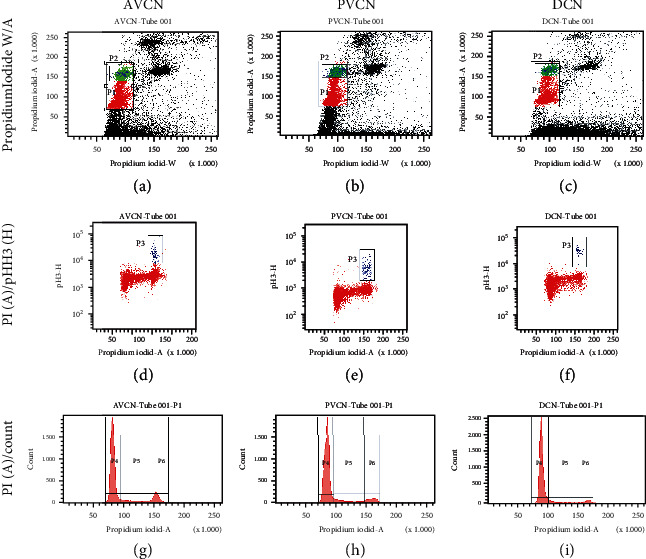
Representative illustration of the flow cytometry analysis with propidium iodide and anti-phospho-histone-H3 from AVCN, PVCN, and DCN NSCs. (a–c) Population of vital cells (P1) in the plot according to width by the area of propidium iodide's fluorescence intensity. P2 shows the population with double DNA content of the G2 and M phases. (d–f) Plot of the population of viable cells gated according to propidium iodide (width) by phospho-histone-H3 (height). P3 shows the population of viable cells with double DNA content—simultaneously stained positively with anti-phospho-histone-H3. (g–i) Histogram of the propidium iodide fluorescence (height) in the viable cell population for quantifying the cell cycle: P4 corresponds to the G0/G1 phase, P5 corresponds to the S phase, and P6 corresponds to the G2 /M phase.

For further validation of the findings obtained in the neurosphere assay, additional flow cytometric analyses were carried out on the cell cycle and the mitotic rate based on the phospho-histone-H3 population (Figure 7).

**Figure 8:**
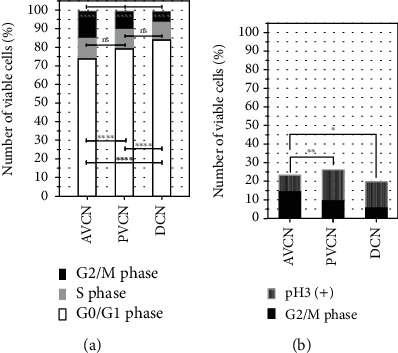
Quantification of flow cytometry analyses from CN NSCs—divided into AVCN, PVCN, and DCN. (a) Quantification of propidium iodide cytometry populations: G2/M, S, and G0/G1 phase. The significantly largest proportion of cells in the G0/S1 phase was determined in the DCN, and the smallest proportion in the AVCN. There were no significant differences between the fractions in the S phase. The largest proportion of cells in the G2/M phase was found in the AVCN, and the smallest in the DCN. (b) Quantification of the G2/M phase population and the proportion of anti-phospho-histone-H3-positive cells. Within the G2/M phase populations, the PVCN showed the significantly largest proportion of cells in which the mitotic marker phospho-histone-H3 was stained positively. The smallest share was in the AVCN. The bar graphs demonstrate the median values; asterisks indicate the significance level: ^∗^*p* < 0.05, ^∗∗^*p* < 0.005, ^∗∗∗^*p* < 0.001, and ^∗∗∗∗^*p* < 0.0001; *n* = 3.

For this purpose, samples of AVCN, PVCN, and DCN were prepared, and 20,000 events of the viable cell population were analyzed and quantified for each sample (Figure 8). The propidium iodide analysis in the G0/S1 phase showed 14,854 ± 143 cells (74 ± 0.7%) in AVCN, 16,426 ± 628 cells (79 ± 1.4%) in PVCN, and 16,773 ± 157 (85 ± 1.4%) (mean ± SD; *n* = 3). The differences between the subnuclei were significant (*p* < 0.0001). In the S phase, the following were found: 2313 ± 25 cells (11.5 ± 0.1%) in the AVCN, 2229 ± 95.8 cells (10.8 ± 0.7%) in the PVCN, and 1996.7 ± 180.6 cells (9.2 ± 10%) in the DCN (mean ± SD; *n* = 3). The proportions of CN NSCs in the S phase did not show any significant differences between the subnuclei. In the G2/M phase, the following were found: 2852.3 ± 126 cells (14.2 ± 0.6%) in AVCN, 1950.7 ± 59 cells (0.4 ± 0.5%) in PVCN, and 1100 ± 129.2 cells (5.5 ± 0.6%) in DCN (mean ± SD; *n* = 3). The significantly highest proportion of cells in the G2/M phase was found in the AVCN, and the lowest in the DCN (*p* < 0.0001). For further analysis, the proportions of anti-phospho-histone-H3-positive (*α*-pHH3+) cells in the G2/M phase populations were determined. The following were found: 254.3 ± 53.7 cells (8.9 ± 1.5%) in the AVCN, 321.3 ± 68.3 cells (16.5 ± 4%) in the PVCN, and 152.3 ± 17.8 cells in the DCN (14.1 ± 2.9%) (mean ± SD; *n* = 3). The significantly largest proportion of *α*-pHH3-positive cells was found in PVCN (*p* = 0.0027), and the lowest in AVCN (*p* = 0.036).

## 4. Discussion

In the auditory system, as in other areas of the central nervous system, quiescent neurogenic niches have been identified. In the CN, equivalent to other regions, it was shown that the highest neurogenic potential can be found in an early postnatal phase but persists until the adult age [16]. There is a critical period after birth in which external factors, such as auditory deprivation or injuries, have a pronounced influence on the regions' neurogenic development [26]. However, specific plastic effects can be observed until adulthood.

In this study, it was found that in the CN, the second relay station of the auditory pathway, there is a neurogenic niche in all subnuclei. However, the neurogenic potential differed significantly between the subdivisions. In the experiments, it was possible to isolate neural stem cells from all CN subnuclei of PND 9 SD rats and to propagate them in free-floating cell cultures. Generated neurospheres and neurosphere-derived single cells from all subdivisions showed typical stem cell criteria: the ability to mitotically divide and self-renew, the ability to develop neural progenitor cells, and multipotency in neuroectodermal maturation [27–29]. The in vitro experiments on self-assembly, self-renewal, and differentiation were carried out in a cascade of experiments. Whole-mount organ cultures were carried out, in which, in addition to neuronal stem cell markers, mitosis was analyzed by BrdU. At the same time, specimens were dissociated and propagated as free-floating cultures. Simultaneous test series were carried out from these cultures, in which neurospheres and single cells were analyzed in parallel. The expression of stem cell markers, the differentiation, and a mitosis assay were carried out in parallel.

### 4.1. Stem Cell Microenvironment

A cell culture system specially adapted to neural stem and progenitor cells had to be used for the experiments described. For the highest possible control of the influencing factors, cells were cultured in serum-free medium (Neurobasal, B-27 supplement, and GlutaMAX) with the mitogens EGF and bFGF (10 ng/ml). These diffusible growth factors are essential for the survival and propagation of neural stem cells in vivo and in vitro [30–33]. They contribute to the embryonic development and patterning [34] of the central nervous system and are considered essential in adult neurogenesis [35].

All differentiation experiments were performed by the withdrawal of these growth factors [36]. Retinoids play an essential role in cell differentiation and maturation of the central nervous system [37]. To mimic these processes in vitro, the serum-free medium and B-27 supplement with retinoic acid were used for cell differentiation. The DIF medium described already has been used successfully in stem cell and differentiation protocols from the precedent studies on CN [14, 16, 17, 38].

For whole-mount organ cultures, an in vitro system on polyester membranes (Transwell® system) was chosen. The adhesive forces of the membrane allow examining anchoring-dependent cells and tissues and closely resemble in vivo conditions [39]. Nutrient solutions can be added basolaterally without mechanically influencing the tissue, and metabolic end products can also be removed. For this reason, the Transwell® system was particularly suitable for the BrdU assay, since short-term medium changes were possible without significantly influencing the tissue. Subsequent immersion fixation of the tissue is also easily possible and, after detachment, the transfer to cryomedia. Using this technique, an investigation of organ cultures was carried out in the experiments described corresponding to the single-cell experiments in liquid cultures.

### 4.2. Mitotic Division and Self-Renewal

To investigate the ability of mitotic division and self-renewal as a stem cell criterion, a neurosphere assay was used, which is a well-established method to analyze a quiescent stem cell potential in vivo in neuronal tissue [28, 40–44]. Indirect methods for stem cell analysis are necessary because there are no distinct markers to identify them [28, 29]. The potential for self-division and self-renewal of the CN subdivisions was therefore analyzed in an experimental series.

During 30 days of free-floating cultures, neurospheres developed in all CN subnuclei, which increased in diameter until the time of evaluation. On average, 8.77 ± 1.76 neurospheres per 1000 single cells formed in AVCN cultures. The PVCN cultures showed the highest potential, with 17.85 ± 1.85 spheres. 5.58 ± 2.87 neurospheres were generated from the DCN cells. There were approximately 0.9% cells with potentially neurogenic potential in the AVCN, 1.8% in the PVCN, and 0.6% in the DCN (relations: PVCN vs.AVCN≙200%; PVCN vs.DCN≙300%) (Figure 1(c)).

To further verify these results of the neurosphere assay, whole-mount organ cultures with BrdU incorporation were investigated in follow-up experiments. The BrdU assay has been established for the analysis of neurogenic potential in tissue in vivo and in vitro [45]. This thymidine analog marks cells in the S phase and can thus indicate neuronal proliferation and self-renewal. The whole-mount organ culture also showed cell division in all CN subnuclei (Figures 5(a)–5(l)). For quantification, the BrdU-labeled cells were calculated in relation to the organ volume (Figure 6). The proliferative potency distribution was very similar to that of the neurosphere assay: the proportion of BrdU-positive cells in PVCN vs. AVCN was 180% and that in PVCN vs. DCN was 300%. Likewise, the PVCN was identified as the CN subdivision with the highest proliferative potential.

To validate the immunohistochemical findings, flow cytometric analyses of CN NSCs from the three subnuclei were performed. Cell cycle studies were carried out using a propidium iodide assay, demonstrating a different distribution of the cell cycle phases (Figure 7). To identify the proliferating cell population, phosphorylated histone H3 was stained, a highly sensitive mitotic marker established for analyzing neurogenic potential in vitro [46]. Phosphorylated histone-H3 has the property of a lower binding capacity to the associated DNA due to a double-negative charge and is a prerequisite for transcription. The BrdU analysis in combination with the pHH3 detection is therefore very specific concerning the mitotic detection of a silent neural stem cell potential [47]. The analysis showed that the significantly largest proportion of pHH3^+^ cells was detected in the area of the PVCN (Figure 8(b)). This confirmed the assumption made in the neurosphere and BrdU assay that PVCN had the greatest neurogenic potential.

To discuss these results in detail, they have to be compared with the capacity to generate neurospheres in other regions of the mammalian brain. They must also be considered in regard to the origin of the subnuclei of the cochlear nucleus. Besides, the analysis techniques applied must also be considered.

In the entire cochlear nucleus, the capacity to form neurospheres was around 30 spheres/1000 cells after three weeks of culture in PND 9 animals [16], which are higher than those in the presented results. In another study, a similar capacity to generate neurospheres was reported in the mouse CN, but younger animals of the PND 3 were examined [13]. Cells from the anteroventral and posteroventral mouse CN formed about 30 spheres/1000 viable cells, and those from the dorsal CN formed about 20 spheres/1000 viable cells. All these numbers are different compared to other regions in the brainstem, for example, the fourth ventricle of adult mice with 0.2 ± 0.06 neurospheres/1000 viable cells after eight days [48]. But even much higher numbers have been previously described in the brainstem [49]. These discrepancies in the sphere quotient can be explained by the distinct neurogenic capacities of individual brain regions and different species. For example, studies have shown that rat-derived neurospheres have a lower growth potential compared to those of mice [50]. The differences in the neurogenic potential can also be explained by the varying culture conditions since in some studies, DMEM/F-12 cell medium was used. In contrast, in the present experiments, Neurobasal medium with growth factors was used, which also has a significant influence on the capacity to form neurospheres [51].

### 4.3. Neural Progenitor Cell Formation

**Figure 9:**
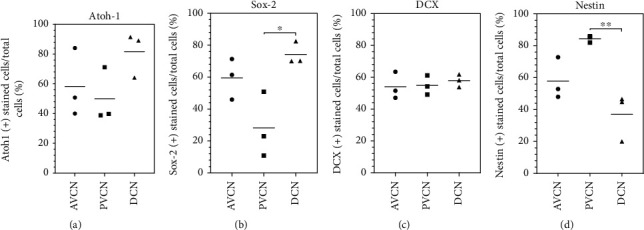
Propagated single cells of all CN subnuclei express neural stem cell markers. Analysis of the distribution of neural stem cell markers of single-cell cultures of CN subnuclei—obtained from neurosphere cultures over 4 weeks. The graphs show the proportions of cells positively marked after 24 h. The *β*-tubulin-positive cells were evaluated as absolute cell numbers. (a) Atoh-1 was stained positively on average in 50–82% of the grown single cells of AVCN, PVCN, and DCN (*n* = 3; mean). There were no significant differences between the subnuclei. (b) On average, 6% of the single cells of the AVCN, 28% of the PVCN, and 74% of the DCN were labeled Sox-2-positive (*n* = 3). The expression of this transcription factor was shown most frequently in dissociated cells of the DCN cultures. (c) The progenitor cell marker doublecortin (DCX) was expressed on average from 54–58% of the plated single cells—without significant differences among the subnuclei (*n* = 3). (d) The neural stem cell marker nestin was expressed on average by 58% of the plated single cells of the AVCN, by 84% of the PVCN, and by 37% of the DCN. The PVCN significantly showed the highest proportion of positively labeled single cells (*n* = 3). Box plots show the median with the upper and lower quartiles, and whiskers mark the upper and lower maximum values; asterisks indicate the significance level: ^∗^*p* < 0.05, ^∗∗^*p* < 0.005, ^∗∗∗^*p* < 0.001, and ^∗∗∗∗^*p* < 0.0001.

To examine the formation of progenitor cells as a further stem cell criterion, immunocytochemical studies on CN neurospheres and of the whole-mount cultures were performed. Typical progenitor cell markers were detected in cells within the neurospheres, but also in emigrated cells. The transcription factor Atoh-1 showed nuclear staining and was found in neurospheres as well as in tissue sections of all CN subdivisions (Figures 2(a)–2(c)). Atoh-1 is responsible for the derivation of the auditory midbrain from the rhombic lip. If Atoh-1 is knocked out, a nonfunctional morphology and missing neuronal connections occur in the CN and the accessory auditory nuclei [52]. This factor is of similar importance to cochlear and hair cell development [53–56]. Therefore, a progenitor cell marker which is exceptionally relevant for the development of the auditory system was detected in all CN subnuclei early postnatally. The further quantification showed that there were no significant differences between the subnuclei in propagated single cells of the CN. Equivalent to this, Atoh-1 also was detected in progenitor cells of the rat inferior colliculus in the early postnatal stage [57]. However, the proportion of positive cells in neurosphere cultures was significantly lower (CN: appr. 50-82% vs. IC: appr. 2.3%; Figure 9(a)). This could be explained by the fact that there is a greater neurogenic potential in the early postnatal CN region than in other brainstem nuclei of the auditory pathway.

Furthermore, the progenitor cell markers Sox-2, doublecortin (DCX), and nestin were detected in the somata of cells in the CN neurospheres and equivalent in whole-mount slices (Figures 5(d)–5(l)). The transcription factor Sox-2 has a vital role in the self-renewal and pluripotency of neural stem cells [58]. Sox-2 is expressed in the development of the neural tube and by proliferating progenitor cells. After proliferation at the maturity stage, the expression is downregulated. Sox-2-expressing neuronal cells are capable of self-renewal and maturation in the forms of the neuroectodermal line [59, 60]. This marker is therefore considered a classic indicator of neural stem and progenitor cells [59]. Sox-2 was detected in proliferating neurospheres of all CN subnuclei and tissue sections of the core areas. In the evaluation of dissociated single cells, the significantly largest proportion of Sox-2 positive cells was determined in the DCN and the smallest percentage in the PVCN. This is in contrast to the neural progenitor cell marker nestin, which was most often positive in PVCN progenitor cells. The intermediate filament nestin is expressed exclusively by neuroepithelial cells in the early stages of CNS development and by neural stem cells [61–63].

Doublecortin is a neuronal migration protein expressed by neural precursor cells, immature embryonic neurons, and adult neural stem cells [64]. After the division of neural stem cells, they express DCX for 2-3 weeks, which is then downregulated [65]. Due to its relative specificity for immature developing neurons, this marker has established itself as a neurogenesis indicator and is often examined with the mitosis marker BrdU.

In summary, two essential stem cell criteria of neural stem cells were shown in all three CN subnuclei: a potential for proliferation and self-renewal—which was expressed both in the formation of neurospheres and in the BrdU incorporation. Furthermore, the formation of neural progenitor cells was demonstrated based on specific expression patterns in the free-floating cultures and histological preparations.

### 4.4. Stem Cell Differentiation

Another fundamental property of neural stem cells is the potential for asymmetric division into progenitor cells and differentiated immature neurons and glial cells [28]. Single cells isolated from cultures of all CN subnuclei were differentiated into the cell types of the neuroectodermal line under withdrawal of growth factors over eight days (Figure 3). Four different cell types were differentiated in these DIF cultures. Multipolar comparatively large cells with rounded somata were even nestin-positive after eight days. These were still in the undifferentiated progenitor cell stage.

Bi- and multipolar cells with spindle-shaped and sometimes roundish somata with slender cell extensions were identified by labeling *β*-III-tubulin as neuronally differentiated cells. These cells formed smaller networks with each other and with glial cells. This type has already been described in NSC cultures of the rat's dorsal vagal complex [49] and other experiments described in literature [28, 29].

Astrocytically differentiated cells had star-shaped extensions that were in contact with other bipolar cells. Immunocytologically, these showed staining of glial fibrillary acidic protein (GFAP). This intermediate filament identifies astroglial cells in vitro [33, 66] and in vivo [67].

The fourth main group in DIF cultures had large somata and spoke-shaped cell branches. Myelin basic protein (MBP) was detected immunocytochemically in the peripheral extensions of these glial cells, thereby identifying them as oligodendrocytes [68]. These also neighbor neural cells in vivo, where they form the myelin sheaths of the axons [69].

The quantification of single-cell DIF experiments had comparable results to other publications in this field: the largest proportion differentiated into the astroglial line, followed by oligodendrocytes. The differentiated neural cells made up the smallest proportion (Figures 3(d)–3(f)) [27, 40, 41]. If one plots the data of the quantification against the individual CN subnuclei, it follows that there were no significant differences. In summary, this shows that progenitor cells were isolated from the tissue of all CN subnuclei. There were substantial differences in the subdivisions' ability to proliferate. Progenitor cells of each of the subdivisions are equally able to differentiate. Accordingly, they do not differ in the potency of maturing.

### 4.5. Spatiotemporal Origin, Migratory Pathway, and Neuronal Specification of the CN Subnuclei

The origin of different rhombomeres might explain the differences between the subnuclei during development. The cochlear nucleus is derived from the rhombomere r2-r5 domain [12]. The PVCN arises from rhombomere 4, as well as the DCN to a lesser degree. Furthermore, the ventral nucleus of the lateral lemniscus and olivocochlear neurons also originates from r4. The neurons derived from r4 contribute more to inhibitory GABAergic and glycinergic neurons than to excitatory glutamatergic neurons [70]. The PVCN also arises from r3 and the DCN from r5, shown by fate analyses in the mouse [71]. In contrast, the r3 region only generates neurons of the AVCN, which are Atoh-1-positive cells [14]. Atoh-1 is a basic helix-loop-helix transcription factor and is essential for the development of cells in the peripheral and central portions of the auditory system [54]. Another publication described two different Atoh-1-dependent rhombic-lip migratory streams. The cranial one contributes to cells in the lateral lemniscus and the cerebellar nuclei, and the caudal one to the cochlear nucleus and the cochlear granule neuron [55]. Taking this knowledge into account, it can be assumed that the differences in origin from distinct regions of the rhombic lip and the variation in expressing important differentiation factors contribute to the different postnatal neurogenic potential of the subnuclei of the cochlear nucleus.

### 4.6. Limitations of the Study

There may be some limitations within the present study. The results about a neurogenic potential in the CN were obtained in vitro based on immunohistochemical examinations and cell culture analyses. Effects of transdifferentiation or a phenotypic modulation of the neural stem and progenitor cells cannot be demonstrated in such a model. Further analyses based on a lineage tracing model could be useful in the future. The present study focused primarily on the possible neurogenic potency within the CN subnuclei at a certain age in the animal model. This model served the purpose of testing and comparing a fundamental neurogenic potential in this specific constellation. Furthermore, the present results were examined using a particular animal model. Further knowledge could in the future be gained from analyzing different species. The additional study of age-related effects would also gain essential insights into the neurogenesis of this brain stem nucleus.

## 5. Conclusions

In conclusion, the results show that all cells with the complete cardinal stem cell criteria are present in the subnuclei of the CN, which provides further insight into the neurogenic potential of this auditory brainstem nucleus. This knowledge might be useful for the development of regenerative strategies, since for such therapeutic approaches, a high neurogenic potential would be beneficial for the replacement of injured or degenerated neural tissue [72]. For example, it could be important for regeneration processes or a neurogenic restructuring after iatrogenic influence such as the implantation of brain stem implants [73, 74]. For this approach, it is of particular importance that there is postnatal neurogenic potential in all CN subnuclei. Therefore, it would be conceivable that both afferent and efferent structures of the CN could be influenced in a potentially regenerative manner, e.g., by exogenously applied substances. Consequently, all functional units (afferent and efferent) of the CN would thus be able to undergo particular neurogenic restructuring or regeneration processes.

## Data Availability

The data used to support the findings of this study are included in the article.

## References

[1] Illing R. B. (2001). Activity-dependent plasticity in the adult auditory brainstem. *Audiology & Neuro-Otology*.

[2] Moore J. K., Niparko J. K., Miller M. R., Linthicum F. H. (1994). Effect of profound hearing loss on a central auditory nucleus. *The American Journal of Otology*.

[3] Kandel E., Schwartz J., Jessell T., Siegelbaum S., Hudspeth A. J. (2012). *Principles of Neural Science*.

[4] Fitzakerley J. L., Schweitzer L. (2003). Morphology of neurons cultured from subdivisions of the mouse cochlear nucleus. *Cell and Tissue Research*.

[5] Campagnola L., Manis P. B. (2014). A map of functional synaptic connectivity in the mouse anteroventral cochlear nucleus. *Journal of Neuroscience: The Official Journal of the Society for Neuroscience*.

[6] Liberman M. C. (1993). Central projections of auditory nerve fibers of differing spontaneous rate, II: posteroventral and dorsal cochlear nuclei. *The Journal of Comparative Neurology*.

[7] ALTMAN J. (1962). Are new neurons formed in the brains of adult mammals?. *Science*.

[8] Deng W., Aimone J. B., Gage F. H. (2010). New neurons and new memories: how does adult hippocampal neurogenesis affect learning and memory?. *Nature Reviews. Neuroscience*.

[9] Sng J., Lufkin T. (2012). Emerging stem cell therapies: treatment, safety, and biology. *Stem Cells International*.

[10] Roccio M., Senn P., Heller S. (2020). Novel insights into inner ear development and regeneration for targeted hearing loss therapies. *Hearing Research*.

[11] Hyakumura T., McDougall S., Finch S., Needham K., Dottori M., Nayagam B. A. (2019). Organotypic cocultures of human pluripotent stem cell derived-neurons with mammalian inner ear hair cells and cochlear nucleus slices. *Stem Cells International*.

[12] Gunewardene N., Crombie D., Dottori M., Nayagam B. A. (2016). Innervation of Cochlear Hair Cells by Human Induced Pluripotent Stem Cell- Derived Neurons In Vitro. *Stem Cells International*.

[13] Volkenstein S., Oshima K., Sinkkonen S. T. (2013). Transient, afferent input-dependent, postnatal niche for neural progenitor cells in the cochlear nucleus. *Proceedings of the National Academy of Sciences of the United States of America*.

[14] Rak K., Wasielewski N. V., Radeloff A. (2011). Isolation and characterization of neural stem cells from the neonatal rat cochlear nucleus. *Cell and Tissue Research*.

[15] Martin M. R., Rickets C. (1981). Histogenesis of the cochlear nucleus of the mouse. *The Journal of Comparative Neurology*.

[16] Rak K., Völker J., Frenz S., Scherzed A., Radeloff A., Hagen R. (2013). Dynamic changes of the neurogenic potential in the rat cochlear nucleus during post-natal development.

[17] Rak K., Völker J., Jürgens L. (2015). Cochlear nucleus whole mount explants promote the differentiation of neuronal stem cells from the cochlear nucleus in co-culture experiments. *Brain Research*.

[18] Shepard A. R., Scheffel J. L., Yu W.-M. (2018). Relationships between neuronal birthdates and tonotopic positions in the mouse cochlear nucleus. *The Journal of Comparative Neurology*.

[19] Sekiya T., Viberg A., Kojima K. (2012). Trauma-specific insults to the cochlear nucleus in the rat. *Journal of Neuroscience Research*.

[20] Wu C., Martel D. T., Shore S. E. (2016). Increased synchrony and bursting of dorsal cochlear nucleus fusiform cells correlate with tinnitus. *Journal of Neuroscience: The Official Journal of the Society for Neuroscience*.

[21] Freemyer A., Neal C., Nelson-Brantley J., Staecker H., Durham D. (2019). Early onset region and cell specific alterations of doublecortin expression in the CNS of animals with sound damage induced hearing loss. *IBRO Reports*.

[22] Han K.-H., Mun S.-K., Sohn S., Piao X.-Y., Park I., Chang M. (2019). Axonal sprouting in the dorsal cochlear nucleus affects gap-prepulse inhibition following noise exposure. *International Journal of Molecular Medicine*.

[23] van Zwieten G., Jahanshahi A., van Erp M. L. (2019). Alleviation of tinnitus with high-frequency stimulation of the dorsal cochlear nucleus: a rodent study. *Trends in Hearing*.

[24] Martel D. T., Pardo-Garcia T. R., Shore S. E. (2019). Dorsal cochlear nucleus fusiform-cell plasticity is altered in salicylate-induced tinnitus. *Neuroscience*.

[25] Schindelin J., Arganda-Carreras I., Frise E. (2012). Fiji: an open-source platform for biological-image analysis. *Nature Methods*.

[26] Trune D. R. (1982). Influence of neonatal cochlear removal on the development of mouse cochlear nucleus: I. Number, size, and density of its neurons. *The Journal of Comparative Neurology*.

[27] Kempermann G., Gage F. H. (1999). New nerve cells for the adult brain. *Scientific American*.

[28] Gage F. H. (2000). Mammalian neural stem cells. *Science*.

[29] Sohur U. S., Emsley J. G., Mitchell B. D., Macklis J. D. (2006). Adult neurogenesis and cellular brain repair with neural progenitors, precursors and stem cells. *Philosophical Transactions of the Royal Society of London. Series B, Biological Sciences*.

[30] Mason I. (2007). Initiation to end point: the multiple roles of fibroblast growth factors in neural development. *Nature Reviews. Neuroscience*.

[31] Vescovi A. L., Reynolds B. A., Fraser D. D., Weiss S. (1993). bFGF regulates the proliferative fate of unipotent (neuronal) and bipotent (neuronal/astroglial) EGF-generated CNS progenitor cells. *Neuron*.

[32] Gritti A., Cova L., Parati E. A., Galli R., Vescovi A. L. (1995). Basic fibroblast growth factor supports the proliferation of epidermal growth factor-generated neuronal precursor cells of the adult mouse CNS. *Neuroscience Letters*.

[33] Murphy M., Drago J., Bartlett P. F. (1990). Fibroblast growth factor stimulates the proliferation and differentiation of neural precursor cells in vitro. *Journal of Neuroscience Research*.

[34] Vaccarino F. M., Schwartz M. L., Raballo R. (1999). Erratum: changes in cerebral cortex size are governed by fibroblast growth factor during embryogenesis. *Nature Neuroscience*.

[35] Maric D., Fiorio Pla A., Chang Y. H., Barker J. L. (2007). Self-renewing and differentiating properties of cortical neural stem cells are selectively regulated by basic fibroblast growth factor (FGF) signaling via specific FGF receptors. *Journal of Neuroscience: The Official Journal of the Society for Neuroscience*.

[36] Belenguer G., Domingo-Muelas A., Ferrón S. R., Morante-Redolat J. M., Fariñas I. (2016). Isolation, culture and analysis of adult subependymal neural stem cells. *Differentiation*.

[37] Jacobs S., Lie D. C., DeCicco K. L. (2006). Retinoic acid is required early during adult neurogenesis in the dentate gyrus. *Proceedings of the National Academy of Sciences of the United States of America*.

[38] Rak K., Wasielewski N., Radeloff A. (2011). Growth behavior of cochlear nucleus neuronal cells on semiconductor substrates. *Journal of Biomedical Materials Research Part A*.

[39] Kim J. H., Sun W., Han D. W., Lim D.-J., Lee J. (2015). Induced neural stem cells have protective effects on cortical neuronal cells in vitro. *Neurological Sciences*.

[40] Gage F. H., Ray J., Fisher L. J. (1995). Isolation, characterization, and use of stem cells from the CNS. *Annual Review of Neuroscience*.

[41] Gage F. H., Coates P. W., Palmer T. D. (1995). Survival and differentiation of adult neuronal progenitor cells transplanted to the adult brain. *Proceedings of the National Academy of Sciences of the United States of America*.

[42] Palmer T. D., Ray J., Gage F. H. (1995). FGF-2-responsive neuronal progenitors reside in proliferative and quiescent regions of the adult rodent brain. *Molecular and Cellular Neurosciences*.

[43] Cooper-Kuhn C. M., Kuhn H. G. (2002). Is it all DNA repair?: methodological considerations for detecting neurogenesis in the adult brain. *Brain Research. Developmental Brain Research*.

[44] Tropepe V., Sibilia M., Ciruna B. G., Rossant J., Wagner E. F., van der Kooy D. (1999). Distinct neural stem cells proliferate in response to EGF and FGF in the developing mouse telencephalon. *Developmental Biology*.

[45] Wojtowicz J. M., Kee N. (2006). BrdU assay for neurogenesis in rodents. *Nature Protocols*.

[46] Bordiuk O. L., Smith K., Morin P. J., Semënov M. V. (2014). Cell proliferation and neurogenesis in adult mouse brain. *PloS One*.

[47] Mori T., Wakabayashi T., Ogawa H., Hirahara Y., Koike T., Yamada H. (2013). Increased histone H3 phosphorylation in neurons in specific brain structures after induction of status epilepticus in mice. *PLoS One*.

[48] Weiss S., Dunne C., Hewson J. (1996). Multipotent CNS stem cells are present in the adult mammalian spinal cord and ventricular neuroaxis. *The Journal of Neuroscience*.

[49] Charrier C., Coronas V., Fombonne J. (2006). Characterization of neural stem cells in the dorsal vagal complex of adult rat by in vivo proliferation labeling and in vitro neurosphere assay. *Neuroscience*.

[50] Svendsen C. N., Skepper J., Rosser A. E., ter Borg M. G., Tyres P., Ryken T. (1997). Restricted growth potential of rat neural precursors as compared to mouse. *Brain Research. Developmental Brain Research*.

[51] Dictus C., Tronnier V., Unterberg A., Herold-Mende C. (2007). Comparative analysis of in vitro conditions for rat adult neural progenitor cells. *Journal of Neuroscience Methods*.

[52] Maricich S. M., Xia A., Mathes E. L. (2009). Atoh1-lineal neurons are required for hearing and for the survival of neurons in the spiral ganglion and brainstem accessory auditory nuclei. *Journal of Neuroscience: The Official Journal of the Society for Neuroscience*.

[53] Mulvaney J., Dabdoub A. (2012). Atoh1, an essential transcription factor in neurogenesis and intestinal and inner ear development: function, regulation, and context dependency. *Journal of the Association for Research in Otolaryngology*.

[54] Bermingham N. A., Hassan B. A., Price S. D. (1999). Math1: an essential gene for the generation of inner ear hair cells. *Science*.

[55] Wang V. Y., Rose M. F., Zoghbi H. Y. (2005). Math1 expression redefines the rhombic lip derivatives and reveals novel lineages within the brainstem and cerebellum. *Neuron*.

[56] Chen P., Johnson J. E., Zoghbi H. Y., Segil N. (2002). The role of Math1 in inner ear development: uncoupling the establishment of the sensory primordium from hair cell fate determination. *Development*.

[57] Völker J., Engert J., Völker C. (2019). Isolation and characterization of neural stem cells from the rat inferior colliculus. *Stem Cells International*.

[58] Rizzino A. (2009). Sox2 and Oct-3/4: a versatile pair of master regulators that orchestrate the self-renewal and pluripotency of embryonic stem cells by functioning as molecular rheostats. https://www.ncbi.nlm.nih.gov/pmc/articles/PMC2794141/.

[59] Ellis P., Fagan B. M., Magness S. T. (2004). SOX2, a persistent marker for multipotential neural stem cells derived from embryonic stem cells, the embryo or the adult. *Developmental Neuroscience*.

[60] Komitova M., Eriksson P. S. (2004). Sox-2 is expressed by neural progenitors and astroglia in the adult rat brain. *Neuroscience Letters*.

[61] Lendahl U., Zimmerman L. B., McKay R. D. (1990). CNS stem cells express a new class of intermediate filament protein. *Cell*.

[62] Kalyani A., Hobson K., Rao M. S. (1997). Neuroepithelial stem cells from the embryonic spinal cord: isolation, characterization, and clonal analysis. *Developmental Biology*.

[63] Hu Z., Tao L., Liu Z., Jiang Y., Deng X. (2019). Identification of neural stem cells from postnatal mouse auditory cortex in vitro. *Stem Cells and Development*.

[64] Couillard-Despres S., Winner B., Schaubeck S. (2005). Doublecortin expression levels in adult brain reflect neurogenesis. *The European Journal of Neuroscience*.

[65] Brown J. P., Couillard-Després S., Cooper-Kuhn C. M., Winkler J., Aigner L., Kuhn H. G. (2003). Transient expression of doublecortin during adult neurogenesis. *The Journal of Comparative Neurology*.

[66] Eng L. F., Ghirnikar R. S., Lee Y. L. (2000). Glial fibrillary acidic protein: GFAP-thirty-one years (1969-2000). *Neurochemical Research*.

[67] Korzhevskii D. E., Otellin V. A., Grigor’ev I. P. (2005). Glial fibrillary acidic protein in astrocytes in the human neocortex. *Neuroscience and Behavioral Physiology*.

[68] Compston A., Zajicek J., Sussman J. (1997). Glial lineages and myelination in the central nervous system. *Journal of Anatomy*.

[69] Bradl M., Lassmann H. (2010). Oligodendrocytes: biology and pathology. *Acta Neuropathologica*.

[70] Di Bonito M., Narita Y., Avallone B. (2013). Assembly of the auditory circuitry by a Hox genetic network in the mouse brainstem. *PLoS Genetics*.

[71] Farago A. F., Awatramani R. B., Dymecki S. M. (2006). Assembly of the brainstem cochlear nuclear complex is revealed by intersectional and subtractive genetic fate maps. *Neuron*.

[72] Horner P. J., Gage F. H. (2000). Regenerating the damaged central nervous system. *Nature*.

[73] Sahyouni R., Chang D. T., Moshtaghi O., Mahmoodi A., Djalilian H. R., Lin H. W. (2017). Functional and histological effects of chronic neural electrode implantation. *Laryngoscope Investigative Otolaryngology*.

[74] Otto S. R., Moore J., Linthicum F., Hitselberger W., Brackmann D., Shannon R. V. (2012). Histopathological analysis of a 15-year user of an auditory brainstem implant. *The Laryngoscope*.

